# Association between overweight/obesity and multidrug-resistant tuberculosis

**DOI:** 10.17843/rpmesp.2023.401.12138

**Published:** 2023-03-30

**Authors:** Jesus Peinado, Leonid Lecca, Judith Jiménez, Roger Calderón, Rosa Yataco, Mercedes Becerra, Megan Murray

**Affiliations:** 1 Partners in Health, Lima, Peru. Partners in Health Lima Peru; 2 School of Medicine, Faculty of Health Sciences, Universidad Peruana de Ciencias Aplicadas, Lima, Peru. Universidad Peruana de Ciencias Aplicadas School of Medicine Faculty of Health Sciences Universidad Peruana de Ciencias Aplicadas Lima Peru; 3 Partners in Health, Boston, USA. Partners in Health Boston USA; 4 Harvard Medical School, Boston, USA. Harvard Medical School Boston USA

**Keywords:** Tuberculosis, Overweight, Obesity, Multidrug Resistance, Peru

## Abstract

**Objective.:**

To evaluate the association between overweight/obesity and multidrug resistance in patients with and without a history of tuberculosis treatment.

**Materials and methods.:**

Cross-sectional study of secondary data from a tuberculosis cohort, which included anthropometric and drug-sensitivity testing data at the baseline visit of patients with and without previous tuberculosis treatment.

**Results.:**

We evaluated 3,734 new cases and 766 with a history of having received treatment for tuberculosis. Overweight/obesity was not associated with multidrug resistance in patients with a history of tuberculosis treatment, with a prevalence ratio of 0.97 and a 95% confidence interval of 0.68-1.38.

**Conclusions.:**

Overweight/obesity is not associated with multidrug resistance in tuberculosis. Overweight/obesity is a dynamic process that may influence the relationship between the immune system and the metabolic system.

## INTRODUCTION

Tuberculosis (TB) affects 8.9 to 11.0 million people and is the cause of death of 1.2 to 1.4 million people globally ^1^. Multidrug-resistant TB (MDR-TB) has a higher rate of treatment failure compared to sensitive TB ^2^. MDR-TB is present in 3.5% of new TB cases and 18% of previously treated cases, which causes 230,000 deaths worldwide ^3^.

Peru is one of the countries from the Latin America and Caribbean (LAC) region with the highest rates of MDR-TB, with an incidence of 9.6 per 100,000 inhabitants, and about 1,205 incident cases per year ^4^. The incidence of MDR-TB has been unusually high in Peru for decades and the factors involved in the higher number of cases in Peru (1.5 times more MDR-TB cases than in other LAC countries) are unknown ^5^.

The emergence of MDR-TB is associated with the amplification of antibiotic resistance patterns through incomplete treatment and transmission in the most vulnerable populations ^6^. The factors that increase the risk of MDR-TB are family contact with MDR-TB diagnosis ^7^, co-infection with human immunodeficiency virus (HIV), heavy tobacco smoking, previous incarceration, history of TB ^8^, and type 2 diabetes *mellitus* (T2DM) ^9^.

On the other hand, the epidemic of overweight and obesity has doubled since 1980. In 2018, about 2 to 5 billion adults were overweight, and 30% of this population was obese ^10^. Like MDR-TB, Peru is one of the LAC countries with the highest overweight rate (38.8%), which is above the regional average (34.4%). In Peru, the prevalence of obesity has increased from 9% in 1975 to 13.8% in men and 23.3% in women in 2010; and up to 23.8% in people aged 30 to 59 years from 2013 to 2014 ^11^.

The immune and the metabolic systems work in synergy because they evolved from common structures, with adipose tissue maintaining the same lineage as the immune cells ^12^. Malnutrition, defined as a body mass index (BMI) under 16, is associated with sputum culture non-conversion within the first four months of MDR-TB treatment ^13^. In addition, a BMI under 18 is a risk factor for developing MDR-TB compared to patients with sensitive TB who have a BMI greater than or equal to 18 ^14^. However, evidence is scarce on whether BMI higher than 25 (overweight/obese) would alter the homeostasis of the metabolic immune axis ^16^.

The main factors affecting the tissue distribution of drugs are BMI, regional blood flow and the affinity of the drug for plasma proteins ^17^. The biomedical literature states that tissue blood flow decreases in obese individuals, and cytochrome P450-2E1 activity increases; however, the effect of overweight/obesity on glomerular filtration is unknown, which motivates the application of weight-standardized maintenance doses to correct for differences in drug clearance ^18^. This is relevant in drugs with a low threshold of pharmacokinetic variation such as rifampicin, one of the main anti-TB drugs where, as a rule of thumb, the dose increases in overweight/obese patients ^19^. Early findings showed that T2DM affected the pharmacokinetics of anti-TB drugs, that blood concentrations of isoniazid and rifampicin were lower in patients with TB and T2DM and that T2DM had an effect on the absorption rate and volume of distribution of rifampicin in patients with TB and T2DM ^19^; however, these statements were later questioned, as the real cause of all these effects was the increased weight of patients with T2DM and TB ^20^.

Low plasma concentrations of isoniazid and rifampicin are associated with increased therapeutic failure and relapse; overweight/obesity affects the concentration-time curve for isoniazid ^21^. Evidence suggests that low plasma concentrations of drugs with high pharmacokinetic variability, such as rifampicin, are involved in the development of MDR-TB ^22^. Weight gain could alter metabolic rates, as occurs in the systemic clearance of other antimicrobials ^23^, and could play a role in modifying plasma concentrations of low pharmacokinetic threshold drugs and be a key determinant for the development of MDR-TB^24^. Therefore, this study aims to evaluate the association between overweight/obesity and multidrug resistance in patients with and without a history of TB treatment.

**KEY MESSAGES**

Motivation for the study. The reason for the increase in cases of multidrug-resistant tuberculosis in Peru compared to the rest of Latin America is unknown. There are factors such as poverty and malnutrition expressed as overweight and obesity that could contribute to the high number of cases.

Main findings. No association was found between overweight/obesity and multidrug-resistant tuberculosis. An association was found between having a history of precious treatment for tuberculosis and multidrug-resistant tuberculosis; however, this association was not be modified by overweight/obesity.

Implications. More studies are needed to understand the joint behavior of the two epidemics facing Peru.

## MATERIALS AND METHODS

### Study type

Analytical, observational, cross-sectional study conducted through a secondary analysis of the database of the cohort “Epidemiology of multidrug-resistant Tuberculosis in Peru” (EPI), which is registered in the ClinicalTrials.gov platform under the code NCT00676754. This cohort was carried out by Socios En Salud Peru branch.

### Study population and sample

We included data from all index cases of the EPI study with positive culture for *Mycobacterium tuberculosis*; data from household contacts were not included. The EPI study population included patients with TB who were recruited in healthcare facilities of the Ministry of Health (MINSA) in 24 districts of the Health Directorates of Lima Centro and Lima Este. We used patient data from June 2008 to June 2014.

### Sample size calculation

The statistical program G*Power version 3.1.9.6 (© 2021 Heinrich-Heine-Universität Düsseldorf, Germany) was used to calculate the sample size. A proportion of MDR-TB in persons with normal BMI of 10% ^5^, a proportion of MDR-TB in overweight/obese persons of 17.4% ^25^, a precision of 0.05, a power of 95% and a sample size ratio of 1 were used as parameters. We obtained a sample size of at least 972 persons.

### Procedures

We considered the data of all participants who met the inclusion criteria: those who had a positive smear or culture, agreed to take the survey, provided sputum and blood samples, and had taken a chest X-ray.

### Data Collection

*Questionnaires*

Baseline data (baseline visit) of index cases regarding demographic characteristics, signs and symptoms of active TB, socioeconomic status indicators, height and weight measurements (measured on weekly calibrated scales), comorbidities (including T2DM), harmful habits (smoking tobacco and drinking alcohol), history of TB treatment and previous incarceration were included. Chest X-ray results were interpreted by two TB experts.

*Biological samples*

Results from bacteriological culture (Petroff method in Löwenstein-Jensen medium), first-line drug sensitivity test (Löwenstein-Jensen method), pyrazinamide susceptibility test (Wayne method), and second-line drug panel (agar plate method) were included.

### Variables

BMI was categorized into underweight (<18.5), normal (18.5-24.9), overweight (25-29.9) and obese (30 or more). Anti-TB drug sensitivity test results were categorized into rifampicin monoresistance and isoniazid and rifampicin resistance (MDR). The dependent variable was MDR-TB, while the independent variable was overweight/obesity (BMI ≥25.0 at enrollment). The covariates were: age categorized as <18, 18-24, 25-44 and 45 or older, sex, history of having received anti-TB treatment (registered by the physician at enrollment), HIV co-infection determined by ELISA test and a confirmatory test, self-report of T2DM, harmful habits (smoking tobacco and drinking alcohol) and previous incarceration. All variables were registered by the nurse at enrollment during the anamnesis. Likewise, we used the poverty score as a variable, which was defined according to its definition in the original study ^26^.

### Statistical analysis

First, data were extracted from the original EPI study using OpenMRS (open-source electronic medical record system). Stata version 16 (Stata Corporation, College Station, Texas, USA) was used to process and analyze the data. The demographic characteristics of the participants were described using absolute frequencies and percentages for qualitative variables; measures of central tendency (mean, median, range) and dispersion (standard deviation and interquartile range) were used for quantitative variables. On the other hand, the proportions of the dependent and independent variables and the covariates were compared using the two-tailed chi-square test and the Mann-Whitney test. Each test was selected based on the evaluated assumptions (normal distribution, quantitative data or categorical data). A two-tailed significance level of less than 0.05 was used as a reference to reject or accept the null hypothesis. Finally, the association between the dependent and independent variables was estimated by calculating the crude (PR) and adjusted (aPR) prevalence ratios with their respective 95% confidence intervals (CI) using a Poisson regression with log link and robust variances. In order to calculate the aPR, an initial model was constructed with the variables associated with the event of interest; then we added the predictor variables that were independently associated with the event of interest. We used a p-value of less than 0.05 in the Wald test to determine whether the association was statistically significant.

### Ethical Aspects

This study was approved by the Ethics Committee of the Universidad Peruana Cayetano Heredia (code 66894). The original study was approved by the bioethics committee of the National Institute of Health and the Harvard School of Public Health, Boston, USA.

## RESULTS

We evaluated 4500 cases, of which 1149 had negative solid-media cultures for *Mycobacterium tuberculosis* at their baseline visit. The final study population consisted of 3351 culture-positive cases. The median age was 28 years with a range of 15 to 94 years, 62.3% were male, 5.2% had previous incarceration, 2.7% reported heavy social smoking, and 44.2% reported heavy social drinking.

Of the 3351 cases, 15.1% had a BMI greater than or equal to 25, 17.1% had a history of TB treatment, 3.5% had HIV coinfection, 5.6% reported having T2DM, and 33.6% had some type of resistance: monoresistance, polyresistance and resistance to isoniazid and rifampicin (MDR) (Table 1).

**Table 1:** Characteristics of the study population.

Characteristics	n (%)
Age (years)	
Younger than 18	225 (6.7)
18-24	1057 (31.5)
25-44	1317 (39.3)
45 or older	752 (22.5)
Sex	
Female	1264 (37.7)
Male	2087 (62.3)
Poverty	
Low	1128 (34.8)
Moderate	1053 (32.5)
High	1063 (32.7)
Previous incarceration	
No	3144 (94.8)
Yes	172 (5.2)
Body mass index	
Low weight (<18.5)	463 (14.0)
Normal (18.5-24.9)	2342 (70.9)
Overweight/Obesity (≥25.0)	500 (15.1)
Tobacco smoking	
Doesn’t smoke	3182 (97.3)
Social/heavy	90 (2.7)
Alcohol consumption	
Doesn’t drink	1788 (55.8)
Social/heavy	1418 (44.2)
History of tuberculosis	
No	2772 (82.9)
Yes	570 (17.1)
Coinfection with HIV	
Negative	3182 (96.5)
Positive	116 (3.5)
Self-reported diagnosis of T2DM	
No	3126 (94.4)
Yes	185 (5.6)
Multidrug-resistant	
No	2150 (84.1)
Yes	406 (15.9)
Rifampicin monoresistance	
No prior treatment	23 (0.9)
Received prior treatment	7 (1.2)
Both populations	30 (0.9)

HIV: human immunodeficiency virus, T2DM: type 2 diabetes *mellitus*.

On the other hand, 15.9% were found to have MDR-TB. When comparing the proportions of covariates and MDR-TB, we found differences in age by category (p=0.007), previous incarceration (p=0.041), and social to heavy tobacco smoking (p=0.010). On the other hand, the difference between MDR-TB and overweight/obesity was not statistically significant (Table 2). We assessed the proportion of MDR-TB in patients with and without a history of TB treatment according to the demographic characteristics and medical history. In this regard, we found differences between not having a history of TB treatment and social/heavy tobacco smoking (p=0.016). We also found differences between having a history of TB treatment and age (p=0.004) and sex (p=0.001) (Table 3).

**Table 2:** Bivariate analysis between multidrug-resistant tuberculosis and sociodemographic characteristics, body mass index, harmful habits, and medical history in the study population.

Characteristics	MDR-TB		p-value ^a^
	No	Yes	
Age (years) (n=2556)			
Median (RIC)	28 (15‒94)	28 (16‒85)	0.058 ^b^
Younger than 18	155 (7.2)	22 (5.4)	0.007
18-24	686 (31.9)	150 (37.0)	
25-44	834 (38.8)	171 (42.1)	
45 or older	475 (22.1)	63 (15.5)	
Sex (n=2556)			
Male	1310 (60.9)	259 (63.8)	0.277
Female	840 (39.1)	147 (36.2)	
Poverty score (n=2481)			
Low	723 (34.7)	139 (34.8)	0.479
Moderate	676 (32.5)	140 (35.1)	
High	683 (32.8)	120 (30.1)	
Prior incarceration (n=2534)			
No	2020 (94.9)	374 (92.4)	0.041
Yes	109 (5.1)	31 (7.6)	
Body mass index (n=2524)			
Low weight (<18,5)	293 (13.8)	59 (14.8)	0.574
Normal (18,5-24,9)	1509 (71.0)	287 (71.9)	
Overweight/Obesity (≥25,0)	323 (15.2)	53 (13.3)	
Tobacco smoking (n=2495)			
Doesn’t smoke	2,049 (97.5)	375 (95.2)	0.010
Social/heavy	52 (2.5)	19 (4.8)	
Alcohol consumption (n=2452)			
Doesn’t drink	1163 (56.3)	211 (54.7)	0.554
Social/heavy	903 (43.7)	175 (45.3)	
History of tuberculosis (n=2551)			
No	1855 (86.5)	261 (64.3)	0.001
Yes	290 (13.5)	145 (35.7)	
Coinfection with HIV (n=2521)			
Negative	2048 (96.6)	385 (96.0)	0.552
Positive	72 (3.4)	16 (4.0)	
Self-reported diagnosis of T2DM (n=2528)			
No	2005 (94.4)	385 (95.5)	0.339
Yes	120 (5.6)	18 (4.5)	

MDR-TB: multidrug-resistant tuberculosis, IQR: interquartile range, HIV: human immunodeficiency virus, T2DM: type 2 diabetes mellitus.

a two-tailed chi-square test, ^b^ Mann-Whitney test.

**Table 3:** Bivariate analysis between multidrug-resistant tuberculosis and sociodemographic variables, harmful habits, and medical history by type of previous TB treatment.

Characteristics	No previous history of TB			History of TB		
	No	MDR-TB	p-value ^a^	No	MDR-TB	p-value ^a^
Age (years) (n=2551)						
Younger than 18	147 (7.9)	18 (6.9)	0.119	7 (2.4)	4 (2.8)	0.004
18-24	631 (34.0)	105 (40.2)		54 (18.6)	45 (31.0)	
25-44	682 (36.8)	96 (36.8)		150 (51.7)	75 (51.7)	
45 or older	395 (21.3)	42 (16.1)		79 (27.3)	21 (14.5)	
Saxo (n=2551)						
Male	1078 (58.1)	165 (63.2)	0.117	229 (79.0)	94 (64.8)	0.001
Female	777 (41.9)	96 (36.8)		61 (21.0)	51 (35.2)	
Poverty score (n=2481)						
Low	604 (33.5)	94 (36.7)	0.324	119 (42.4)	45 (31.5)	0.091
Moderate	586 (32.6)	87 (34.0)		90 (32.0)	53 (37.0)	
high	611 (33.9)	75 (29.3)		72 (25.6)	45 (31.5)	
Previous incarceration (n=2534)						
No	1781 (96.8)	248 (95.0)	0.140	239 (82.7)	126 (87.5)	0.196
Yes	59 (3.2)	13 (5.0)		50 (17.3)	18 (12.5)	
Body mass index (n=2522)						
Low weight (<18,5)	238 (12.9)	34 (13.2)	0.847	55 (19.2)	25 (17.6)	0.452
Normal (18,5-24,9)	1323 (72.1)	188 (73.2)		184 (64.1)	99 (69.7)	
Overweight/Obesity (≥25,0)	275 (15.0)	35 (13.6)		48 (16.7)	18 (12.7)	
Tobacco smoking (n=2495)						
Doesn’t smoke	1779 (97.8)	242 (95.3)	0.016	270 (95.7)	133 (95.0)	0.728
Social/heavy	40 (2.2)	12 (4.7)		12 (4.3)	7 (5.0)	
Alcohol consumption (n=2452)						
Doesn’t drink	1056 (59.0)	144 (58.1)		107 (38.8)	67 (48.6)	0.057
Social/heavy	734 (41.0)	104 (41.9)	0.780	169 (61.2)	71 (51.4)	
Coinfection with HIV (n=2520)						
Negative	1775 (96.9)	246 (95.7)	0.323	272 (94.8)	139 (96.5)	0.414
Positive	57 (3.1)	11 (4.3)		15 (5.2)	5 (3.5)	
Self-reported diagnosis of T2DM (n=2528)						
No	1735 (94.4)	247 (95.0)	0.690	270 (94.1)	138 (96.5)	0.282
Yes	103 (5.6)	13 (5.0)		17 (5.9)	5 (3.5)	

TB: tuberculosis; MDR-TB: multidrug-resistant tuberculosis; HIV: human immunodeficiency virus; T2DM: type 2 diabetes mellitus.

a two-tailed chi-square test.

The multivariate analysis showed that MDR-TB was not associated with being underweight (aPR: 1.05; 95%CI: 0.74-1.49 in patients with treatment history; aPR: 0.87; 95%CI: 0.59-1.28 in patients with no treatment history) nor with overweight/obesity (aPR: 0.96; 95%CI: 0.68-1.38 in patients with treatment history; aPR: 0.88; 95%CI: 0.57-1.38 in patients with no treatment history).

## DISCUSSION

Few studies explore the relationship between the TB epidemic and the overweight/obesity epidemic, particularly regarding MDR-TB; with the aim of implementing strategies to help eradicate TB. We assessed the association between overweight/obesity and MDR-TB in patients with and without a history of TB (cases with a history of TB and new TB cases). Our results show that there is no association between overweight/obesity and MDR-TB, i.e., overweight/obesity was not associated with MDR-TB in new patients without a history of TB treatment or in patients with a history of TB treatment.

Song *et al*. found an association between new MDR-TB cases and being overweight in data collected from 2004 to 2019 ^25^, which differs from our results. Earlier studies on this subject reported that overweight/obesity was associated with low risk of active TB, although they did not evaluate MDR-TB ^27^^,^^28^; their findings also are different from ours. The difference between our results and those from other studies could be due to genetic variations related to pharmacogenetics, which was proposed by Pasipanodya *et al*. ^24^. This variability could explain the high rates of MDR-TB in the Peruvian territory, being one of the countries with the highest rates in the LAC region for several decades ^5^. Another possible for this difference is the type of overweight/obesity, Peru being the country with the highest percentage of energy derived from carbohydrate consumption (62.9%), unlike other countries in the region, where obesity is due to the high consumption of fats and proteins ^29^.

Another possible cause may be the fact that overweight/obese women account for 5-6% of the population in the other studies, whereas in our study they represent 19-20% of the total sample. This agrees with the study by Woolcott *et al*., who conducted a cross-sectional analysis of 31,549 people from the National Center of Food and Nutrition of Peru and reported that the crude prevalence of obesity is 11.3% for men and 20.7% for women and varies according to the altitude of residence, which complicates overweight and obesity ^30^. Besides, the aforementioned studies only evaluated new cases, whereas our study included old and new TB cases. Considering these subpopulations, we found that overweight/obesity would not modify the association between previous TB treatment and MDR-TB ^8^^,^^31^.

Variables classically associated with MDR-TB such as HIV coinfection, T2DM and poverty did not have statistically significant differences in this study. It is possible that we did not find differences between HIV infection and MDR-TB because HIV is more frequent in specific populations that may have not been represented in our study. Other studies reported an association between HIV coinfection and MDR-TB, particularly primary MDR-TB, in populations with higher HIV rates, such as Africa or Asia ^32^. In our study, data regarding T2DM diagnosis was based on self-reports, and given that T2DM is an almost asymptomatic disease in its early stages, it is likely that it was not accurately diagnosed ^33^, which may explain why we did not find differences between T2DM and MDR-TB. Additionally, no differences were found between poverty and MDR-TB, which may be due to the type of instrument we used ^34^^,^^35^, given that the original study recruited participants from poor districts of Lima, it is possible that the number of non-poor people is too small to determine a statistically significant difference.

Our study has several important limitations. The cross-sectional design prevents us from establishing causality between the variables, and we could only determine their association. The BMI categories were based on single cut-off points for all age groups, without considering that for people aged 60 years or older, who made up 9.5% of the sample, the cut-off points could be different. The history of having received treatment for TB was self-reported during the anamnesis. This, together with the fact that there is no information available to confirm whether the patient was cured or not after the treatment, could cause a recall bias. In addition, T2DM diagnosis was also self-reported, which does not allow diagnostic confirmation and prevents establishing absolute glycemic values. Overweight/obesity changes over time, this condition may have changed between diagnosis and treatment. We did not collect information regarding weight before enrollment, nor during follow-up, this is an important limitation, since, this variable could increase with age. On the other hand, we found that only 2.3% of the participants were obese, which is why we added the overweight population (12.8%). This lack of cases with nutritional disorders could limit the statistical power to evaluate their association with MDR-TB. The most appropriate design would have been a large cohort during several years of observation and data collection, not only for BMI but also for other nutritional parameters such as waist-to-hip ratio.

We conclude that there is no association between overweight/obesity and anti-TB drug resistance in patients with a history of TB treatment.
