# Supplementary material for: Association between overweight/obesity and multidrug-resistant tuberculosis
Source: Rev Peru Med Exp Salud Publica. 2023 Mar 30;40(1):59–66. doi: 10.17843/rpmesp.2023.401.12138 (PMC10953666; doi:10.17843/rpmesp.2023.401.12138)
Supplement: Translation

# Translation: Asociación entre sobrepeso/obesidad y tuberculosis multidrogoresistente

**Authors:** Jesus Peinado, Leonid Lecca, Judith Jiménez, Roger Calderón, Rosa Yataco, Mercedes Becerra, Megan Murray

**Affiliations:** 1 Socios en Salud Sucursal Perú, Lima, Perú.; 2 Escuela de Medicina, Facultad de Ciencias de la Salud, Universidad Peruana de Ciencias Aplicadas, Lima, Perú.; 3 Partners in Health, Boston, Estados Unidos.; 4 Harvard Medical School, Boston, Estados Unidos.

**Keywords:** Tuberculosis, Sobrepeso, Obesidad, Resistencia a Multidrogas, Perú

## Abstract

**Objetivo.:**

Evaluar la asociación entre el sobrepeso/obesidad y la multidrogoresistencia en pacientes con y sin antecedentes de tratamiento para tuberculosis.

**Materiales y métodos.:**

Estudio transversal realizado a través de un análisis secundario de la base de datos de una cohorte de tuberculosis, que incluyó datos de pruebas antropométricas y pruebas de sensibilidad a drogas en la visita basal de pacientes con y sin tratamiento previo para tuberculosis.

**Resultados.:**

Se evaluaron 3,734 casos nuevos y 766 con antecedente de haber recibido tratamiento para tuberculosis. El sobrepeso/obesidad no se asoció a la multidrogoresistencia en pacientes con antecedente de tratamiento para tuberculosis, mostrando una razón de prevalencia de 0,97 con un intervalo de confianza al 95% de 0,68-1,38.

**Conclusiones.:**

El sobrepeso/obesidad no está asociado a la multidrogoresistencia en tuberculosis; siendo el sobrepeso/obesidad un proceso dinámico que puede influir en las relaciones entre el sistema inmune y el sistema metabólico.

## INTRODUCCIÓN

La tuberculosis (TB) afecta de 8,9 a 11,0 millones de personas y es la causa de muerte de 1,2 a 1,4 millones de personas a nivel global ^1^. La TB en su forma multidrogoresistente (TB-MDR) posee una mayor tasa de fracasos al tratamiento comparado con la TB sensible ^2^. El 3,5% de los casos nuevos de TB y el 18% de los casos previamente tratados, presentan TB-MDR, la cual, causa 230,000 muertes alrededor del mundo ^3^.

Perú es uno de los países que pertenecen a la región de Latinoamérica y el Caribe (LAC) con mayores cifras de TB-MDR, donde destaca una incidencia de 9,6 por 100,000 habitantes, y cerca de 1,205 casos incidentes anuales ^4^. Esta incidencia de la TB-MDR es inusualmente alta en el Perú desde hace décadas y los factores que estarían involucrados en un mayor número de casos en el territorio peruano (1,5 veces más casos de TB-MDR respecto a otros países de LAC) son desconocidos ^5^. 

La aparición de la TB-MDR está asociada a la amplificación de los patrones de resistencia a través del tratamiento incompleto y la transmisión en las comunidades más vulnerables ^6^. Los factores que incrementan el riesgo de TB-MDR son el contacto familiar con diagnóstico de TB-MDR ^7^, la coinfección por el virus de la inmunodeficiencia humana (VIH), fumar tabaco a un grado intenso, antecedente de encarcelamiento, antecedente de TB ^8^ y padecer de diabetes mellitus tipo 2 (DM2) ^9^. 

Por otro lado, la epidemia del sobrepeso y la obesidad se ha duplicado desde 1980. En 2018, cerca de 2 a 5 mil millones de adultos tenían sobrepeso, siendo que el 30% de esta población eran obesos ^10^. Al igual que la TB-MDR, Perú es uno de los países de LAC con mayores cifras de sobrepeso, registrándose un sobrepeso poblacional del 38,3%, cifra que está por encima del promedio regional (34,4%). En Perú, la prevalencia de obesidad ha incrementado de 9% en 1975 a 13,8% en los hombres y 23,3% en las mujeres en el año 2010; y hasta 23,8% en las personas de 30 a 59 años para el periodo de 2013 a 2014 ^11^.

El sistema inmune y el sistema metabólico trabajan sinérgicamente debido a que han evolucionado de estructuras comunes, siendo que el tejido adiposo mantiene el mismo linaje que las células del sistema inmune ^12^. La desnutrición, expresada como un bajo índice de masa corporal (IMC) (menor a 16) está asociada a la no conversión del cultivo de esputo dentro de los primeros cuatro meses de tratamiento en pacientes con TB-MDR ^13^. Además, un IMC menor a 18 es un factor de riesgo para desarrollar TB-MDR comparado con aquellos pacientes con TB sensible que tienen un IMC mayor o igual a 18 ^14^,^15^. Sin embargo, es escasa la evidencia sobre si el incremento del IMC más allá de 25 (sobrepeso/obesidad) alteraría la homeostasis del eje inmune metabólico ^16^. 

Los principales factores que afectan la distribución tisular de los fármacos son el IMC, el flujo sanguíneo regional y la afinidad del fármaco por las proteínas plasmáticas ^17^. La literatura biomédica señala que existe una reducción en el flujo sanguíneo tisular en los individuos obesos, y un aumento en la actividad del citocromo P450-2E1; sin embargo, el efecto del sobrepeso/obesidad sobre la filtración glomerular no está bien establecido, lo que motiva a aplicar dosis de mantenimiento normalizada según el peso, para corregir las diferencias en el aclaramiento del fármaco ^18^. Esto resulta relevante en los fármacos con bajo umbral de variación farmacocinética como la rifampicina, una de las principales drogas anti-TB donde por regla empírica, se aumenta la dosis en pacientes con sobrepeso/obesidad ^19^. Los primeros hallazgos indicando que la DM2 afectaba la farmacocinética de las drogas anti-TB, que las concentraciones sanguíneas de isoniazida y rifampicina eran más bajas en los pacientes con TB y DM2 y que existiría un efecto de la DM2 sobre la constante de velocidad de absorción y el volumen de distribución de la rifampicina en los pacientes con TB y DM2 ^19^, fueron posteriormente cuestionados, ya que la causa real de todos estos efectos era el aumento de peso de los pacientes con DM2 y TB ^20^.

Las bajas concentraciones plasmáticas de isoniazida y rifampicina se asocian con un mayor fracaso terapéutico y recaída; y el sobrepeso/obesidad afecta la curva de concentración-tiempo para la isoniazida ^21^. La evidencia sugiere que las bajas concentraciones plasmáticas de los medicamentos con gran variabilidad farmacocinética, como la rifampicina, están involucradas en el desarrollo de la TB-MDR ^22^. El incremento de peso podría alterar las tasas metabólicas, tal como sucede en el aclaramiento sistémico de otros antimicrobianos ^23^, y podría desempeñar un rol en la modificación de las concentraciones plasmáticas de drogas de bajo umbral farmacocinético y ser un determinante clave para el desarrollo de la TB-MDR ^24^. Por lo tanto, el objetivo del estudio fue evaluar la asociación entre el sobrepeso/obesidad y la multidrogoresistencia en pacientes con y sin antecedentes de tratamiento de TB.

**MENSAJES CLAVE**

Motivación para realizar el estudio. El motivo del aumento de los casos de tuberculosis multidrogoresistente en el Perú en comparación con América Latina no esta bien establecido. Existen factores como la pobreza y la malnutrición expresada en el sobrepeso y obesidad que podrían contribuir a la aparición de un mayor número de casos.

Principales hallazgos. No se encontró asociación entre el sobrepeso/obesidad y la tuberculosis multidrogoresistente. Únicamente, se halló una asociación entre tener antecedente de haber sido tratado por tuberculosis y tuberculosis multidrogoresistente, sin embargo, esta asociación no sería modificada por el sobrepeso/obesidad.

Implicancias. Se necesitan realizar un mayor número de estudios para entender el comportamiento conjunto de las dos epidemias que afronta el Perú.

## MATERIALES Y MÉTODOS

### Tipo de estudio

Estudio analítico, observacional, transversal realizado a través de un análisis secundario de la base de datos de la cohorte «Epidemiología de la Tuberculosis multidrogo resistente en el Perú» (EPI), que se encuentra registrada en la plataforma ClinicalTrials.gov bajo el código NCT00676754. Esta cohorte fue desarrollada por Socios En Salud sucursal Perú. 

### Población de estudio y muestra

En el presente estudio, se utilizaron los datos de todos los casos índices del estudio EPI con cultivo positivo para *Mycobacterium tuberculosis* y no se incluyeron los datos de los contactos domiciliarios. La población del estudio EPI incluyó a pacientes con TB diagnosticados que fueron captados en los establecimientos de salud del Ministerio de Salud (MINSA) de un total 24 distritos de las Direcciones de Salud Lima Centro y Lima Este. El periodo de inclusión de los pacientes fue desde junio de 2008 hasta junio de 2014.

### Cálculo del tamaño de muestra

El cálculo del tamaño de muestra se realizó con el programa estadístico G*Power versión 3.1.9.6 (© 2021 Heinrich-Heine-Universität Düsseldorf, Germany). Como parámetros, se consideró una proporción de TB-MDR en personas con IMC normal del 10% ^5^, una proporción de TB-MDR en personas con sobrepeso/obesidad del 17,4% ^25^, una precisión del 0,05, una potencia del 95% y una razón de tamaños muestrales de 1. Finalmente, se obtuvo un tamaño de muestra de 972 personas como mínimo.

### Procedimientos

Para el presente estudio, se consideraron a todos los datos de los participantes que cumplieron con los criterios de inclusión: pacientes con baciloscopia positiva o con cultivo positivo y pacientes que dieron su consentimiento para completar los cuestionarios, brindaron muestras de esputo y sangre y participaron en la toma de una radiografía de tórax.

### Recolección de datos

*Cuestionarios*

Se incluyeron los datos basales (visita basal) de los casos índices sobre las características demográficas, signos y síntomas de TB activa, indicadores de estado socioeconómico, medida de talla y peso (medidos en balanzas calibradas semanalmente), comorbilidades (incluyendo a la DM2), hábitos nocivos (fumar tabaco y beber alcohol), antecedente de tratamiento para TB y de encarcelamiento. Los resultados de la radiografía de tórax fueron interpretados por dos expertos en TB.

*Muestras biológicas*

Se incluyeron los resultados de los cultivos bacteriológicos (método de Petroff en medio Löwenstein-Jensen), los resultados de las pruebas de sensibilidad a drogas de primera línea (método de Löwenstein-Jensen), la susceptibilidad a pirazinamida (método Wayne), y los resultados del panel de drogas de segunda línea (método de agar en placa).

### Variables

El IMC fue categorizado en bajo peso (<18,5), normal (18,5-24,9), sobrepeso (25-29,9) y obesidad (30 o más). Los resultados de la prueba de sensibilidad a drogas anti-TB fueron clasificados en monoresistencia a rifampicina y resistencia a isoniazida y rifampicina (MDR), siendo la variable dependiente la TB-MDR, mientras que la variable independiente fue el sobrepeso/obesidad (IMC ≥25,0, registrada en el enrolamiento). Por otro lado, las covariables incluidas fueron la edad categorizada en <18, 18-24, 25-44 y 45 o más años; respectivamente, sexo, antecedente de haber recibido tratamiento anti-TB (registrada por el médico en el enrolamiento), coinfección por VIH determinada por prueba ELISA y una prueba confirmatoria, autorreporte de DM2, hábitos nocivos (fumar tabaco y beber alcohol) y antecedente de encarcelamiento. Todas las variables fueron registradas por la enfermera en el enrolamiento durante la anamnesis. Asimismo, se utilizó la variable score de pobreza, cuya definición es idéntica a la del estudio original ^26^.

### Análisis estadístico

Primero, se extrajeron los datos del estudio original EPI utilizando el sistema de información OpenMRS (open-source electronic medical record platform). El procesamiento de datos y el análisis fueron realizados en el programa estadístico Stata versión 16 (Stata Corporation, College Station, Texas, USA). Las características demográficas de los participantes fueron descritas mediante frecuencias absolutas y porcentajes para las variables cualitativas, mientras que, para las variables cuantitativas, se calculó las medidas de tendencia central (media, mediana, rango) y de dispersión (desviación estándar y rango intercuartílico). Por otro lado, se realizó una comparación entre las proporciones de las variables dependiente e independiente, y las covariables mediante la prueba estadística de chi-cuadrado para dos colas y la prueba de Mann-Whitney. Cada prueba fue seleccionada en base a los supuestos evaluados (distribución normal, dato cuantitativo o dato categórico). Se usó de referencia un nivel significancia de dos colas menor de 0,05 para rechazar o aceptar la hipótesis nula. Finalmente, la asociación entre la variable dependiente e independiente se estimó por el cálculo de las razones de prevalencia crudas (RP) y ajustadas (RPa) con sus respectivos intervalos de confianza (IC) al 95% usando una regresión de Poisson con enlace logarítmico y varianzas robustas. Para el cálculo de las RPa, se realizó un modelo inicial con las variables asociadas al evento de interés, luego agregamos al modelo, las variables predictoras que están asociadas de manera independiente al evento de interés. Usamos un valor de p menor a 0,05 del test de Wald para determinar si la asociación era estadísticamente significativa.

### Aspectos éticos

Se obtuvo aprobación del Comité de Ética de la Universidad Peruana Cayetano Heredia (código 66894). El estudio original tuvo aprobación del comité de bioética del Instituto Nacional de Salud y de la Escuela de Salud Pública de Harvard de Boston, Estados Unidos.

## RESULTADOS

Se evaluaron los datos de 4,500 casos, de los cuales, 1,149 casos tuvieron cultivos negativos para *Mycobacterium tuberculosis* en medio sólido en su visita basal. Finalmente, se consideró a una población de estudio de 3,351 casos con cultivo positivo para el análisis. La mediana de edad fue 28 años con un rango de 15 a 94 años, el 62,3% fue de sexo masculino, el 5,2% tuvo antecedente de prisión, el 2,7% reportó fumar de manera social a una forma intensa y el 44,2% reportó beber alcohol de manera social a una forma intensa.

De los 3,351 casos, el 15,1% tenía un IMC mayor o igual a 25, el 17,1% tenía antecedente de tratamiento de TB, el 3,5% tenía coinfección por VIH, el 5,6% reportó tener DM2, y el 33,6% tenía algún tipo de resistencia: monoresistencia, poliresistencia y resistencia a isoniacida y rifampicina (MDR) (Tabla 1).

**Tabla 1:** Características de la población de estudio.

Características	n (%)
Edad (en años)	
Menor a 18	225 (6,7)
18-24	1057 (31,5)
25-44	1317 (39,3)
45 o más	752 (22,5)
Sexo	
Femenino	1264 (37,7)
Masculino	2087 (62,3)
Score de pobreza	
Baja	1128 (34,8)
Mediana	1053 (32,5)
Alta	1063 (32,7)
Antecedente de encarcelamiento	
No	3144 (94,8)
Sí	172 (5,2)
Índice de masa corporal	
Bajo peso (<18,5)	463 (14,0)
Normal (18,5-24,9)	2342 (70,9)
Sobrepeso/Obesidad (≥25,0)	500 (15,1)
Fuma tabaco	
No fuma	3182 (97,3)
Social/intenso	90 (2,7)
Bebe alcohol	
No bebe	1788 (55,8)
Social/intenso	1418 (44,2)
Antecedente de tuberculosis	
No	2772 (82,9)
Sí	570 (17,1)
Coinfección por VIH	
Negativo	3182 (96,5)
Positivo	116 (3,5)
Diagnóstico autorreportado de DM2	
No	3126 (94,4)
Sí	185 (5,6)
Multidrogoresistencia	
No	2150 (84,1)
Sí	406 (15,9)
Monoresistencia a rifampicina	
Sin antecedente de tratamiento	23 (0,9)
Con antecedente de tratamiento	7 (1,2)
Ambas poblaciones	30 (0,9)

VIH: virus de la inmunodeficiencia humana, DM2: diabetes *mellitus* tipo 2.

Por otro lado, se encontró que el 15,9% eran casos con TB-MDR. Al comparar las proporciones de la covariables y TB-MDR, se reportaron diferencias en la edad por categorías (p=0,007), antecedente de encarcelamiento (p=0,041), y fumar tabaco de forma social a intensa (p=0,010). Por otro lado, la TB-MDR no se tuvo diferencias estadísticamente significativas con el sobrepeso/obesidad (Tabla 2). Al evaluar las proporciones de TB-MDR y las características demográficas y los antecedentes clínicos en pacientes con y sin antecedentes de tratamiento para TB, encontramos diferencias en las siguientes variables: fumar tabaco de forma social a intensa (p=0,016) en pacientes sin antecedente de tratamiento para TB, mientras que, en pacientes con antecedente de tratamiento para TB, se encontraron diferencias en las variables edad (p=0,004) y sexo (p=0,001) (Tabla 3).

**Tabla 2:** Análisis bivariado entre tuberculosis multidrogoresistente y características sociodemográficas, índice de masa corporal, hábitos nocivos, y antecedentes clínicos en la población de estudio.

Características	TB-MDR		Valor de p ^a^
	No	Sí	
Edad (en años) (n=2556)			
Mediana (RIC)	28 (15‒94)	28 (16‒85)	0,058 ^b^
Menor a 18	155 (7,2)	22 (5,4)	0,007
18-24	686 (31,9)	150 (37,0)	
25-44	834 (38,8)	171 (42,1)	
45 o más	475 (22,1)	63 (15,5)	
Sexo (n=2556)			
Masculino	1,310 (60,9)	259 (63,8)	0,277
Femenino	840 (39,1)	147 (36,2)	
Score de pobreza (n=2481)			
Baja	723 (34,7)	139 (34,8)	0,479
Mediana	676 (32,5)	140 (35,1)	
Alta	683 (32,8)	120 (30,1)	
Antecedente de encarcelamiento (n=2534)			
No	2020 (94,9)	374 (92,4)	0,041
Sí	109 (5,1)	31 (7,6)	
Índice de masa corporal (n=2524)			
Bajo peso (<18,5)	293 (13,8)	59 (14,8)	0,574
Normal (18,5-24,9)	1,509 (71,0)	287 (71,9)	
Sobrepeso/Obesidad (≥25,0)	323 (15,2)	53 (13,3)	
Fuma tabaco (n=2495)			
No fuma	2,049 (97,5)	375 (95,2)	0,010
Social/intenso	52 (2,5)	19 (4,8)	
Bebe alcohol (n=2452)			
No bebe	1,163 (56,3)	211 (54,7)	0,554
Social/intenso	903 (43,7)	175 (45,3)	
Antecedente de tuberculosis (n=2551)			
No	1,855 (86,5)	261 (64,3)	0,001
Sí	290 (13,5)	145 (35,7)	
Coinfección por VIH (n=2521)			
Negativo	2,048 (96,6)	385 (96,0)	0,552
Positivo	72 (3,4)	16 (4,0)	
Diagnóstico autorreportado de DM2 (n=2528)			
No	2,005 (94,4)	385 (95,5)	0,339
Sí	120 (5,6)	18 (4,5)	

TB-MDR: tuberculosis multidrogoresistente, RIC: rango intercuartílico, VIH: virus de la inmunodeficiencia humana, DM2: diabetes *mellitus* tipo 2.

a prueba chi-cuadrado de dos colas, ^b^ prueba de Mann-Whitney.

**Tabla 3:** Análisis bivariado entre tuberculosis multidrogoresistente y variables sociodemográficas, hábitos nocivos, y antecedentes clínicos por tipo de antecedente de tratamiento de TB.

Características	No antecedente de TB			Antecedente de TB		
	No	TB-MDR	Valor de p ^a^	No	TB-MDR	Valor de p ^a^
Edad (en años) (n=2551)						
Menor a 18	147 (7,9)	18 (6,9)	0,119	7 (2,4)	4 (2,8)	0,004
18-24	631 (34,0)	105 (40,2)		54 (18,6)	45 (31,0)	
25-44	682 (36,8)	96 (36,8)		150 (51,7)	75 (51,7)	
45 o más	395 (21,3)	42 (16,1)		79 (27,3)	21 (14,5)	
Sexo (n=2551)						
Masculino	1,078 (58,1)	165 (63,2)	0,117	229 (79,0)	94 (64,8)	0,001
Femenino	777 (41,9)	96 (36,8)		61 (21,0)	51 (35,2)	
Score de pobreza (n=2481)						
Baja	604 (33,5)	94 (36,7)	0,324	119 (42,4)	45 (31,5)	0,091
Mediana	586 (32,6)	87 (34,0)		90 (32,0)	53 (37,0)	
Alta	611 (33,9)	75 (29,3)		72 (25,6)	45 (31,5)	
Antecedente de encarcelamiento (n=2534)						
No	1,781 (96,8)	248 (95,0)	0,140	239 (82,7)	126 (87,5)	0,196
Sí	59 (3,2)	13 (5,0)		50 (17,3)	18 (12,5)	
Índice de masa corporal (n=2522)						
Bajo peso (<18,5)	238 (12,9)	34 (13,2)	0,847	55 (19,2)	25 (17,6)	0,452
Normal (18,5-24,9)	1,323 (72,1)	188 (73,2)		184 (64,1)	99 (69,7)	
Sobrepeso/Obesidad (≥25,0)	275 (15,0)	35 (13,6)		48 (16,7)	18 (12,7)	
Fuma tabaco (n=2495)						
No fuma	1,779 (97,8)	242 (95,3)	0,016	270 (95,7)	133 (95,0)	0,728
Social/intenso	40 (2,2)	12 (4,7)		12 (4,3)	7 (5,0)	
Bebe alcohol (n=2452)						
No bebe	1,056 (59,0)	144 (58,1)		107 (38,8)	67 (48,6)	0,057
Social/intenso	734 (41,0)	104 (41,9)	0,780	169 (61,2)	71 (51,4)	
Coinfección por VIH (n=2520)						
Negativo	1,775 (96,9)	246 (95,7)	0,323	272 (94,8)	139 (96,5)	0,414
Positivo	57 (3,1)	11 (4,3)		15 (5,2)	5 (3,5)	
Diagnóstico autorreportado de DM2 (n=2528)						
No	1,735 (94,4)	247 (95,0)	0,690	270 (94,1)	138 (96,5)	0,282
Sí	103 (5,6)	13 (5,0)		17 (5,9)	5 (3,5)	

TB: tuberculosis, TB-MDR: tuberculosis multidrogoresistente, VIH: virus de la inmunodeficiencia humana, DM2: diabetes *mellitus* tipo 2.

a prueba chi-cuadrado de dos colas.

En el análisis multivariado, encontramos que la TB-MDR no se asoció al bajo peso (RPa: 1,05; IC 95%: 0,74-1,49 en pacientes con antecedente de tratamiento y RPa: 0,87; IC 95%: 0,59-1,28 en pacientes sin antecedente de tratamiento) ni al sobrepeso/obesidad (RPa: 0,96; IC 95%: 0,68-1,38 en pacientes con antecedente de tratamiento y RPa: 0,88; IC 95%: 0,57-1,38 en pacientes sin antecedente de tratamiento).

## DISCUSIÓN

Existen pocos estudios que exploran la relación entre la epidemia de la TB y la epidemia del sobrepeso/obesidad, sobre todo en la aparición de TB-MDR, con el fin de implementar estrategias para contribuir a eliminar la TB. En nuestro estudio evaluamos la asociación entre el sobrepeso/obesidad y TB-MDR en pacientes con y sin antecedente de TB (casos con historia de TB y casos nuevos de TB). Se encontró que no existe una asociación entre el sobrepeso/obesidad y TB-MDR, es decir, el sobrepeso/obesidad no se asoció a la TB-MDR en pacientes nuevos sin antecedente de tratamiento para TB ni en pacientes con historia de tratamiento para TB.

Nuestros resultados difieren de los resultados reportados por Song *et al.* en un estudio que incluyó datos recogidos durante el periodo de 2004 a 2019. En este estudio, los autores hallaron una asociación entre casos nuevos de TB-MDR y sobrepeso ^25^. Asimismo, nuestro hallazgo es disímil de los primeros estudios realizados sobre el tema, donde se reporta que el sobrepeso/obesidad está asociado a un bajo riesgo de TB activa, aunque no se analiza la TB-MDR ^(^^27^^,^^28^. Una primera explicación de la diferencia de nuestros resultados con los reportados en otros estudios podría basarse en las variaciones genéticas dentro del campo de la farmacogenética, lo cual fue postulado por Pasipanodya *et al*. ^24^. Esta variabilidad contribuiría a explicar las altas tasas de TB-MDR en el territorio peruano, siendo uno de los países con mayores cifras de esta entidad en la región de LAC desde hace varias décadas ^5^. Una segunda explicación es el tipo de sobrepeso/obesidad, siendo Perú, el país que posee el porcentaje más alto de energía derivado del consumo de carbohidratos (62,9%) a diferencia de otros países de la región, donde la obesidad se debe al alto consumo de grasas y proteínas ^29^.

Una tercera explicación deriva del sexo del individuo, ya que, las mujeres con sobrepeso/obesidad son el 5-6% de la población incluida en los estudios, mientras que en nuestro estudio constituye del 19 al 20% del total de la muestra. Este hecho va en concordancia con el estudio de Woolcott *et al.*, los cuales, realizaron un análisis transversal de 31 549 personas del Centro Nacional de Alimentos y Nutrición de Perú y reportaron que la prevalencia bruta de obesidad es del 11,3% para los hombres y 20,7% para las mujeres y varia por la altitud de residencia, lo cual, complejiza el comportamiento del sobrepeso y la obesidad ^30^. Una cuarta explicación, es que Song *et al.* y otros estudios solo evalúan a nuevos casos, mientras que en nuestro estudio se incluyeron a los casos antiguos y nuevos de TB. Considerando a estas subpoblaciones, se encontró que el sobrepeso/obesidad no modificaría la asociación entre el antecedente de tratamiento de TB y la TB-MDR ^8^^,^^31^.

Las variables que clásicamente se asocian a la TB-MDR como la coinfección por el VIH, DM2 y pobreza no tuvieron diferencias estadísticamente significativas en este estudio. La posible razón de no encontrar diferencias entre la infección por el VIH con la TB-MDR, sería que la epidemia de VIH en el territorio peruano está concentrada en poblaciones clave que difieren de la población incluida en el presente estudio, mientras que en otros estudios ^32^ donde se reporta una asociación de coinfección por VIH y TB-MDR, sobre todo para TB-MDR primaria, ocurre en las poblaciones con una epidemia generalizada del VIH en regiones como África y Asia. La probable explicación de no encontrar diferencias entre la DM2 y la TB-MDR; primero, es debido a que la DM2 en nuestro estudio es un dato autorreportado en la anamnesis, y dado que la DM2 es una enfermedad casi asintomática en sus fases iniciales, es probable que no se haya diagnosticado efectivamente ^33^. Adicionalmente, no se encontraron diferencias entre la pobreza y TB-MDR, lo cual puede deberse al tipo de instrumento utilizado para su medición ^34^^,^^35^, dado que el estudio original reclutó a participantes de distritos con mayor pobreza de Lima, es posible que haya poco porcentaje de personas no pobres para determinar una diferencia estadísticamente significativa.

Nuestro estudio tiene varias limitaciones importantes. El diseño transversal del estudio impide establecer causalidad entre las variables, y solo se podría determinar su asociación. Las categorías del IMC fueron calculadas en base a puntos de corte únicos para todos los grupos etarios, sin considerar que para las personas de 60 años o más que conforman el 9,5% de la muestra, los puntos de corte podrían ser diferentes. El antecedente de haber sido tratado de TB es un dato autorreportado durante la anamnesis. Esto aunado a que no se cuenta con información que pueda confirmar si el paciente terminó curado o no curado en su anterior tratamiento, podría introducir un sesgo de memoria. Además, otra variable que es autorreportada y que no permite una confirmación diagnóstica es la DM2, lo que impide establecer valores glicémicos absolutos. El sobrepeso/obesidad es dinámico en el tiempo, puede haber tenido sobrepeso/obesidad todo el tiempo desde que terminó o abandonó el tratamiento de TB anterior o puede haber ganado peso previo al diagnóstico de TB. En este estudio, no hemos recolectado el peso antes del enrolamiento, ni tampoco durante el seguimiento, esta es una limitación importante, ya que, la variable peso aumentaría con la edad. Por otro lado, se encontró que solo el 2,3% de los participantes tenía obesidad, razón por la cual se ha tenido que agregar a la población con sobrepeso (12,8%). Esta falta de casos con desordenes nutricionales, limitaría la potencia estadística para evaluar su asociación con la TB-MDR. El diseño más adecuada hubiese sido una cohorte amplia con varios años de observación y recolección de datos, no solo del IMC sino también de otros parámetros nutricionales como índice cintura-cadera.

Concluimos que no existe una asociación entre el sobrepeso/obesidad y la resistencia a fármacos anti-TB en pacientes con antecedente de tratamiento de TB.
